# Supplementary material for: Targeting EZH2 reactivates a breast cancer subtype-specific anti-metastatic transcriptional program
Source: Nat Commun. 2018 Jun 29;9:2547. doi: 10.1038/s41467-018-04864-8 (PMC6026192; doi:10.1038/s41467-018-04864-8)
Supplement: Supplementary file 1 — Supplementary Information [file 41467_2018_4864_MOESM1_ESM.pdf]

**Supplementary Information**

Targeting EZH2 reactivates a breast cancer subtype-specific anti-metastatic transcriptional program

Hirukawa et al.

Supplementary Figures 1-7

Supplementary Tables 1-3

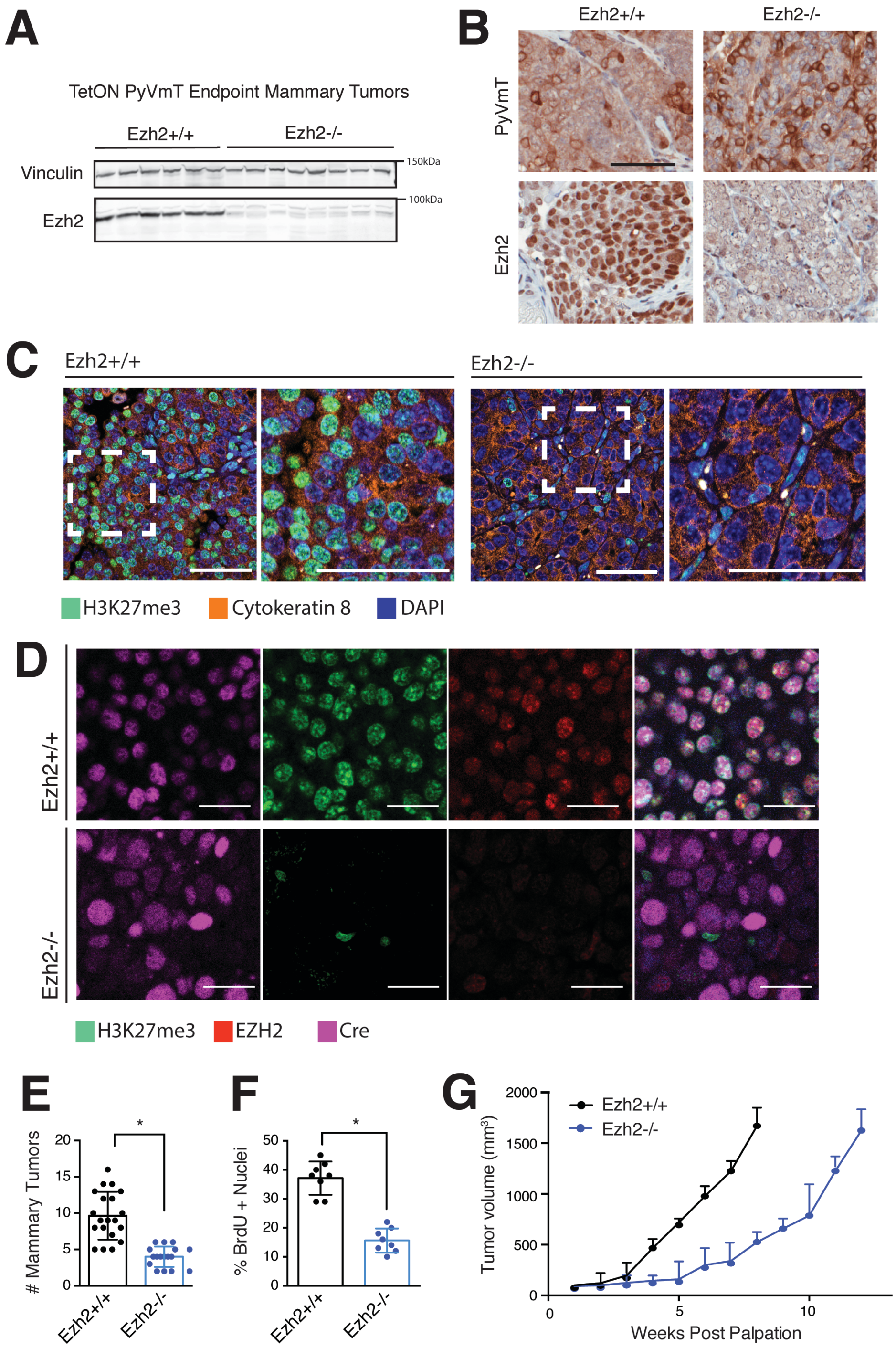


**Supplementary Figure 1.** Deletion of Ezh2 impairs mouse mammary tumorigenesis.

**Supplementary Figure1.** Deletion of Ezh2 impairs mouse mammary tumorigenesis. (A) Immunoblot of endpoint Ezh2+/+ or Ezh2-/- Tet-ON PyVmT tumors assessed for protein levels of Ezh2 and PyVmT. Vinculin was used a loading control. (B) Representative images of paraffin-embedded sections of endpoint Tet-ON PyVmT tumors stained by immunohistochemistry for Ezh2 and PyVmT levels. Scale bars are 300μm. (C) Representative immunofluorescence images of endpoint end point Ezh2+/+ or Ezh2-/- Tet-ON PyVmT tumors stained with $H3K27me^3$, the epithelial marker cytokeratin 8 (CK8) or the nuclear stain DAPI. $H3K27me^3$ is visible in CK8 positive tumour epithelium of the wild type but is restricted to CK8 negative cells in the conditional knock out. Scale cars are 50μm. (D) Representative immunofluorescence images of endpoint end point Ezh2+/+ or Ezh2-/- Tet-ON PyVmT tumors stained with $H3K27me^3$, Ezh2 or Cre. Scale bars are 50μm. (E) Quantification of the total number of mammary tumors per mouse, for each genotype. (F) Percentage of BrdU positive nuclei in endpoint Tet-ON PyVmT tumors. All mice were injected with BrdU (0.05mg/gram) and sacrificed after 2 hours to allow for BrdU incorporation into cells. (G) Total tumor volume of Ezh2+/+ or Ezh2-/- Tet-ON PyVmT tumors. *$p<0.05$, Student's t-test, 2 tailed.

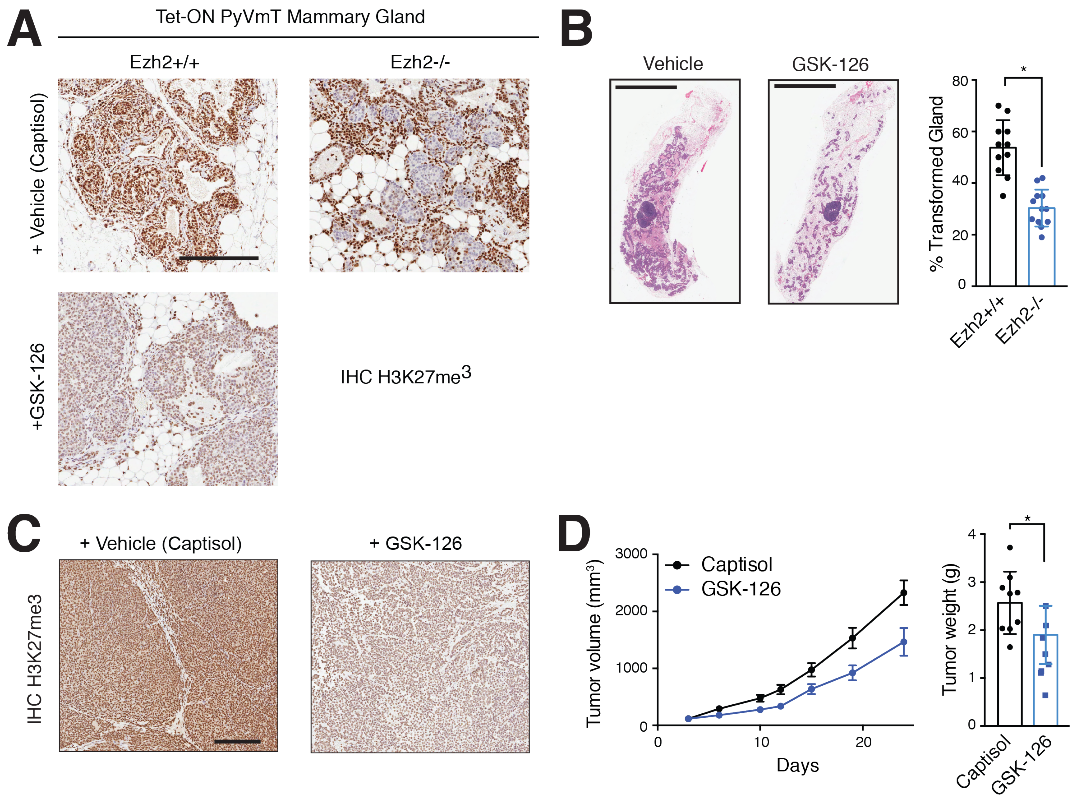


**Supplementary Figure 2.** Pharmacological inhibition of Ezh2 methyltransferase inhibits metastasis *in vivo*. (A) Representative images of paraffin-embedded sections of wild-type Tet-ON PyVmT mice induced with doxycycline for 2 weeks to form hyperplastic lesions and then treated with GSK-126 or vehicle for 4 weeks, or Ezh2 -/- Tet-ON PyVmT mice induced with doxycycline for 6 weeks. Sections were stained to assess global H3K27me$^3$ levels by immunohistochemistry (IHC). Scale bars are all 200μm. (B) Representative images of paraffin-embedded sections of wild-type Tet-ON PyVmT mouse mammary glands stained with hematoxylin and eosin in GSK-126 or vehicle treated mice, as outlined in Figure 2A. Scale bars are all 4mm. Quantification of the percentage of transformed mammary glands between conditions. (C) Paraffin-embedded sections of tumors from PyVmT cells orthotopically injected into the mammary fat pad of FVB hosts, and treated with GSK-126 or vehicle for 3 weeks. Scale bars are all 1mm. (D) Tumor growth of mice treated with GSK-126 or vehicle control. Quantification of tumor weight after 3 weeks of drug treatment.  * $p<0.05$, Student's t-test, 2-tailed.

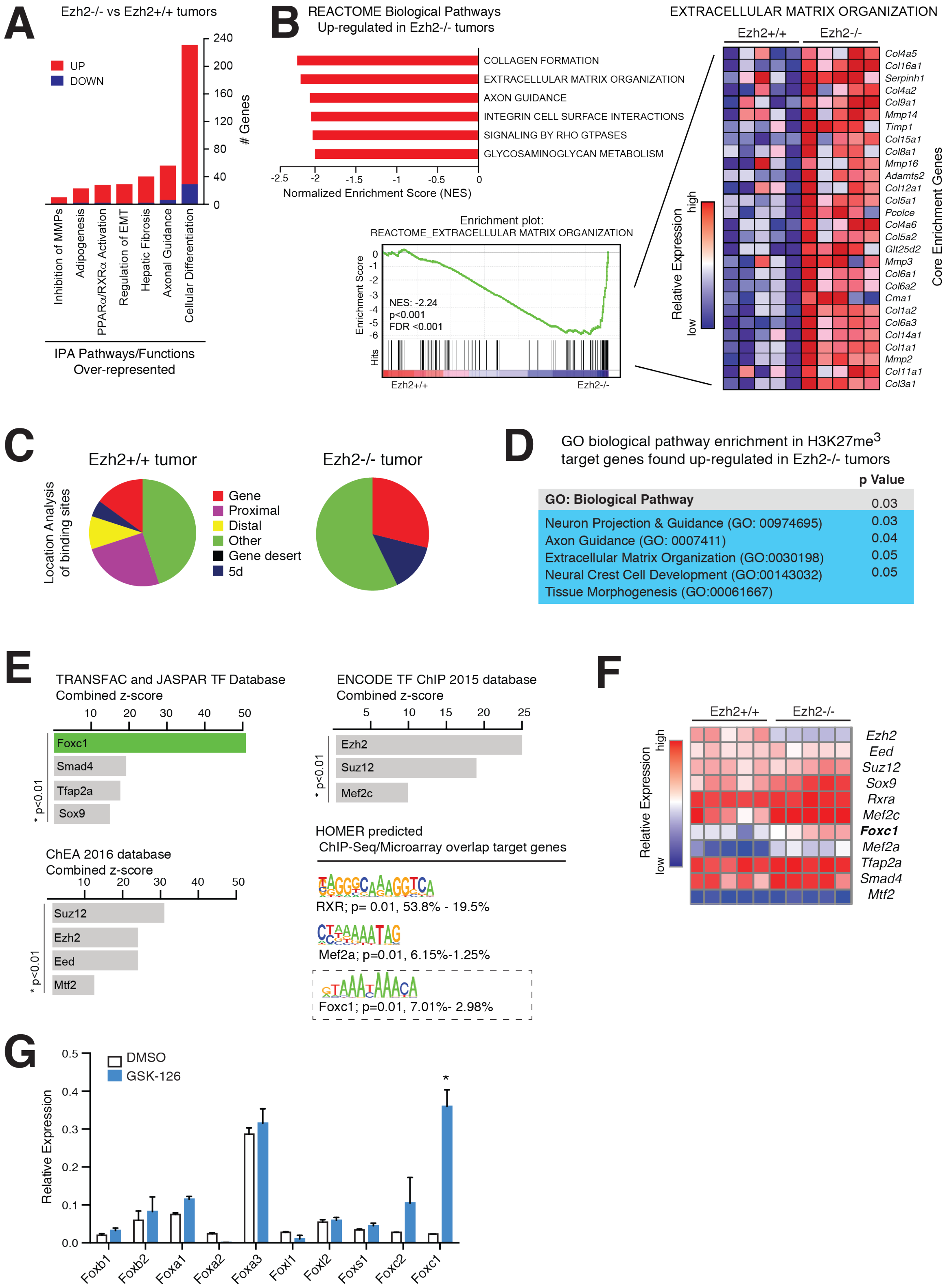


**Supplementary Figure 3.** Functional genomics identifies Foxc1 as a target of H3K27me3 mediated repression.

**Supplementary Figure 3.** Functional genomics identifies Foxc1 as a target of H3K27me$^3$ mediated repression. (A) Schematic of a subset of over-represented pathways and functions identified by IPA analysis of genes found differentially expressed in Ezh2 null tumors. (B) GSEA identification of significantly up-regulated Reactome biological pathways in Ezh2-/- tumors. A representative plot and heatmap illustrating the increased expression of genes associated with ECM organization in Ezh2 null tumors are shown. (C) Pie chart and histogram showing ChIP-seq H3K27me$^3$ mapping across the genome. (D) Gene Ontology (GO) terms most significantly enriched in genes differentially upregulated in Ezh2-/- endpoint tumors, that were also H3K27me$^3$ targets. (E) Transcription factor enrichment for genes up-regulated in the Ezh2-null mammary tumors that were also identified as being targets of H3K27me$^3$ using public predictive or curated databases JASPAR, ENCODE ChIP and ChEA and arranged by their combined z-score. Right panel illustrates *de novo* DNA motif enrichment analysis of gene targets identified between the intersection of ChIP-seq targets and differentially upregulated genes in Ezh2 -/- tumours. Proportion of peaks containing the motifs compared to background are indicated below the transcription factors and p values associated with each motif are shown. * $p<0.01$(F) Heatmap of relative expression levels of upstream transcription factors identified in Supplementary Figure 3 E, in Ezh2 +/+ or Ezh2-/- endpoint Tet ON PyVmT tumours. (G) PyVmT cells treated with GSK-126 (2μM, 72 hours) significantly up-regulated the Forkhead box family member, *Foxc1*, compared to DMSO control treated cells. * $p<0.05$, Student’s t-test, 2-tailed.

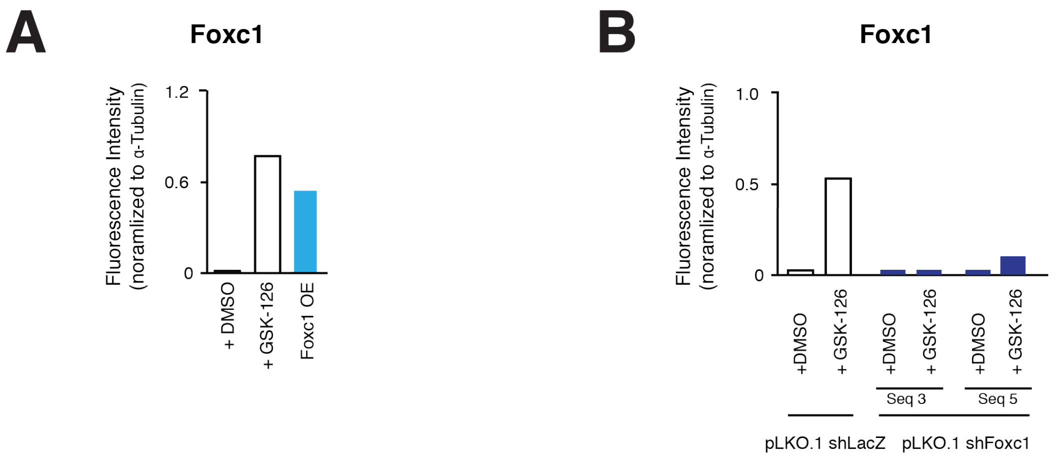


**Supplementary Figure 4**. Quantification of FOXC1 levels. (A) Quantification of fluorescence intensity of FOXC1 in PyVmT cells treated with DMSO, GSK-126 (2μM), or stably expressing exogenous Foxc1 using the LICOR Odyssey system and normalized to the house keeping gene α-Tubulin). (B) Quantification of fluorescence intensity of FOXC1 in DMSO, GSK-126 (2μM) treated in PyVmT cells stably knocking down Foxc1 or LacZ.

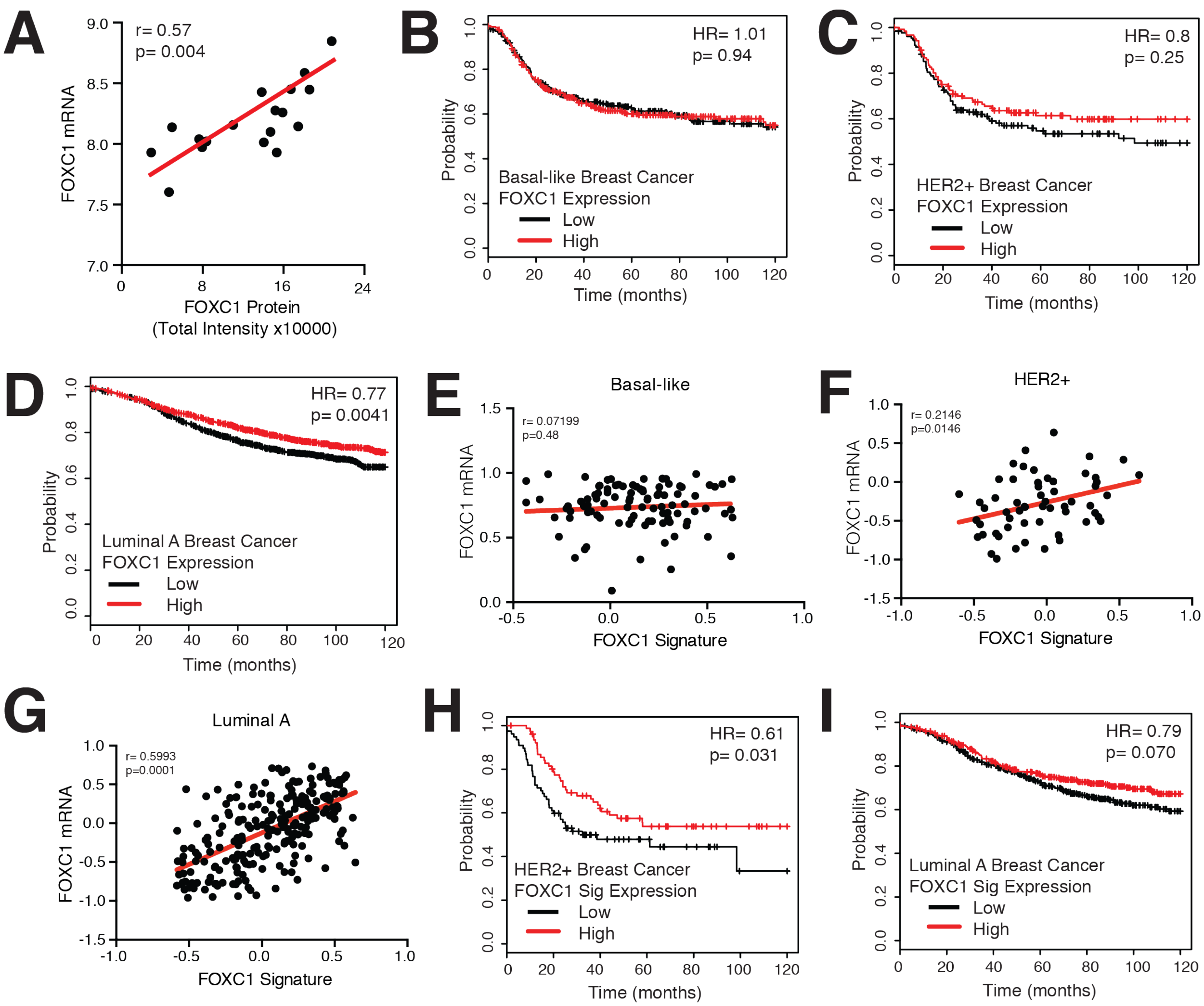


**Supplementary Figure 5.** FOXC1 in molecular subtypes of breast cancer.

**Supplementary Figure 5.** FOXC1 in molecular subtypes of breast cancer. (A) Significant positive correlation between FOXC1 transcript levels derived from microarray profiling of Luminal B patient tumor samples and protein levels as assessed by immunofluorescence staining (r= 0.57, p=0.004). (B) Kaplan-Meier survival curve of relapse free survival of 618 BLBC patients with high or low FOXC1 (no significant difference). (C). Kaplan-Meier survival curve of relapse free survival of 335 HER2+ patients with high or low FOXC1 (no significant difference, but trend towards higher levels of *FOXC1* conferring better outcome, p=0.25). (D) Kaplan-Meier survival curve of relapse free survival of 2504 Luminal A patients with high or low FOXC1 (p=0.004). (E) No correlation between the *FOXC1* gene signature and *FOXC1* transcript levels in Basal-like patients from the TCGA Breast Cancer database (r=0.07, p=0.48). (F) Significant positive correlation between the *FOXC1* gene signature and *FOXC1* transcript levels in HER2+ patients from the TCGA Breast Cancer database (r=0.21, p=0.015). (G) Significant positive correlation between the *FOXC1* gene signature and *FOXC1* transcript levels in Luminal A patients from the TCGA Breast Cancer database (r=0.6, p=0.0001). (H) Kaplan-Meier survival curve of relapse free survival of 335 HER2+ patients with high or low FOXC1 signature (p=0.031). (I) Kaplan-Meier survival curve of relapse free survival of 2504 Luminal A patients with high or low FOXC1 signature (p=0.07).

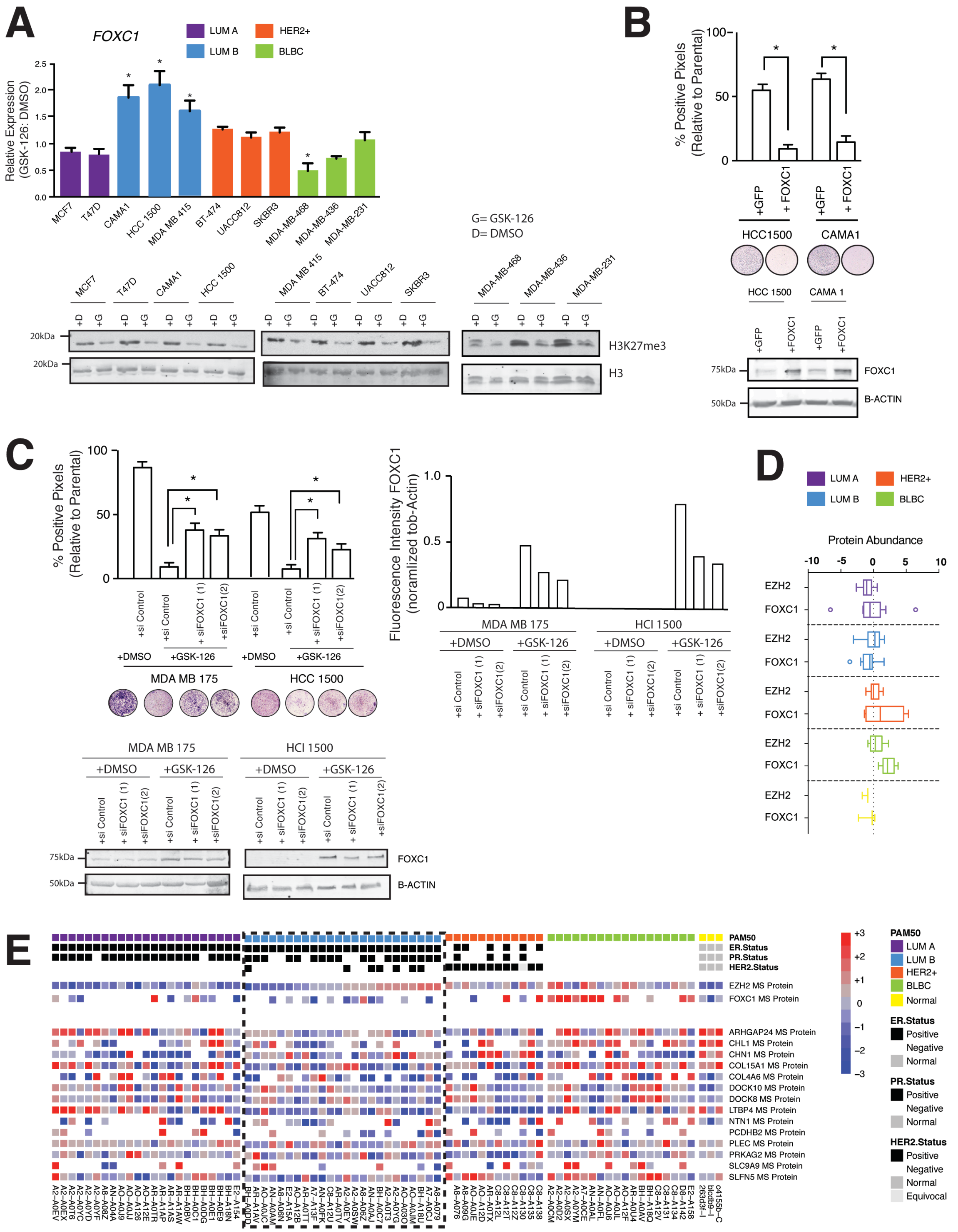


**Supplementary Figure 6.** Specificity of EZH2 mediated repression of FOXC1 across different breast cancer subtypes.

**Supplementary Figure 6**. Specificity of EZH2 mediated repression of FOXC1 across different breast cancer subtypes. (A) qRT-PCR analysis of FOXC1 levels in different breast cancer cells lines following 72 hours of treatment with GSK-126 (2μM) or DMSO (vehicle) for 72 hours. Samples were assayed in triplicate, and *FOXC1* expression was normalized to the housekeeper gene *ACTB*. Lower panel includes immunoblots for total H3 and histone H327me3 from isolated histones of the corresponding human cell lines treated with DMSO or GSK-126 (2μM). (B) Transwell invasion assay of established human Luminal B breast cancer cells expressing exogenous Foxc1 or GFP. Experiments were performed three times, and results are displayed relative to untreated parental cell. Lower panel includes an immunoblot of the FOXC1 levels in cells exogenous expressing FOXC1 or control GFP. (C) Transwell invasion assay of PyVmT cells infected with control or siRNA or two different sequences targeting Foxc1. Cells were pre-treated with DMSO or GSK-126 (2μM) for 72 hours and assayed for their capacity to invade through Matrigel. All assays were performed in triplicate. Immunoblot demonstrates RNAi of FOXC1 in the human cells lines used in the invasion assay and the LI-COR Odyssey software system was used to normalize the signal of FOXC1 to β Actin to assess knock down. (D) Turkey box plots showing median human EZH2 and total FOXC1 or EZH2 protein levels in 77 human tumours representing the PAM50 molecular breast cancer subtype classification along with 3 normal breast tissue samples. Data is from Mertins et al 2016 [1]. (E) Heatmap of mass spectrometry-based proteomics data for EZH2, FOXC1 and 14 out of 23 members of the identified FOXC1 targets. Black dotted box surrounds the Luminal B samples.

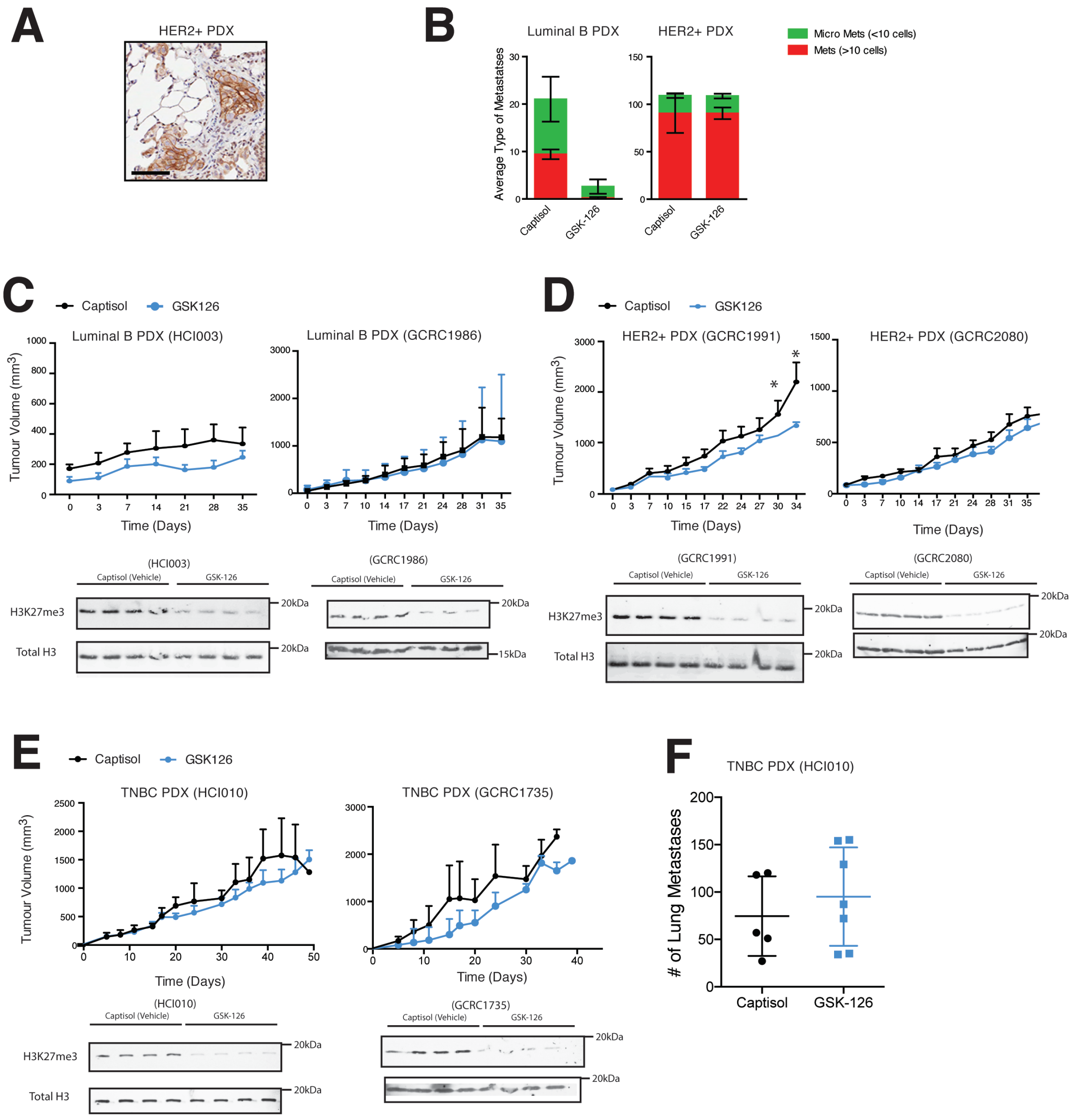


**Supplementary Figure 7.** Specificity of global H3K27me3 reduction across patient derived xenografts representative of different breast cancer subtypes.

**Supplementary Figure 7.** Specificity of global H3K27me3 reduction across patient derived xenografts representative of different breast cancer subtypes. (A) Representative staining of lung lesion for HER2 by immunohistochemistry. Scale bar is 100µm. (B) Quantification of intravascular or extravascular lung lesions in spontaneous lung metastases from a Luminal B PDX (n=5 per condition) or a HER2+ PDX (n=6 per condition) transplanted into a NOG host and treated with GSK-126 or vehicle. (C) Tumor growth of two different Luminal B PDXs treated with GSK-126 or vehicle (n=5 per treatment group). An immunblot of total H3 or H3K27me3 was performed on extracted histones from endpoint tumours from both cohorts to confirm efficacy of GSK-126. (D) Tumor growth of two different HER2+ PDXs treated with GSK-126 or vehicle (n= 6 per treatment group). * $p<0.05$, Student's t-test, 2-tailed. An immunoblot of total H3 or H3K27me3 was performed on extracted histones from endpoint tumours from both cohorts to confirm efficacy of GSK-126. (E) Tumor growth of two different TNBC PDXs treated with GSK-126 or vehicle (n= 6 per treatment group). * $p<0.05$, Student's t-test, 2 tailed. (F) Quantification of lung lesions following the implantation of TNBC (HCI010) into the mammary fat pad, followed by treatment with GSK-126 (n=7) or vehicle (Captisol, n=5). While highly proliferative, TNBC PDX GCRC 1735 was not spontaneously metastatic to the lungs.

## Supplementary Tables

Supplementary Table 1. Human and mouse quantitative RT-PCR primer sequences

| Gene | Sequences | Species |
|---|---|---|
| *Slc9a9* | F-GGTTTACGTCAGGGAAGGATG, R-AGAAGCGGAATCGGTGATTTTT | Mouse |
| *Col4a6* | F-ATCGGATACTCCTTCCTCATGC, R-CCAGGGGAGACTAGGGACTG | Mouse |
| *Col15a1* | F-CCCAGGGAAGAATGGAGAAGT, R-CCAGAGCCTTCAATCTCAAATCC | Mouse |
| *Dbc1* | F-AGCGCCCTATCCCATGTATCA, R-AGCTTGTAGCCCTTATTGCAG | Mouse |
| *Chl1* | F-TCCGCCAGGAAACCTTCAC, R-ACAATGTACTCGCTGATTTTGCT | Mouse |
| *Chn1* | F-GCAGCCAGGAACGTACACTTT, R-ATCAGTCACCAGATCGTGGAT | Mouse |
| *Slfn5* | F-AAGGGAGGAAATGGATACCACA, R-CTCAACCCTGACCACTCCG | Mouse |
| *Dock8* | F-GTGGAAATACGTCCAGTACCTG, R-CGATGGGTCAATCGAGGGTG | Mouse |
| *Slc35f2* | F-CTGGGTACGGTGGACAGTATC, R-GCATCAACGTGTAAACCAGGA | Mouse |
| *Pcdhb16* | F-GGGAACTGAGGCGCTATTCTG, R-TTAGCAAGCATGGCTCTGTATG | Mouse |
| *Ltbp4* | F-CTGGGTGTCGCTATTGGTG, R-GTTGTGACAGATCAAGGGACAT | Mouse |
| *Rgma* | F-AGAGGACCTTATGAGCCAGCA, R-GCAGTGAGTGTAGTTGGGGG | Mouse |
| *Pde3b* | F-AAAGCGCAGCCGGTTACTAT, R-CACCACTGCTTCAAGTCCCAG | Mouse |
| *B Actin* | F-TCCATCATGAAGTGTGACGT, R-GAGCAATGATCTTGATCTTCAT | Mouse |
| *Foxl2* | F-ACAACACCGGAGAAACCAGAC, R-CGTAGAACGGGAACTTGGCTA | Mouse |
| *Foxl1* | F-GAGCAGAGGGTCACACTGAAC, R-CTTCCTGCGCCGATAATTGC | Mouse |
| *Foxc2* | F-GCGTAGCTCGATAGGGCAG, R-AACCCAACAGCAAACTTTCCC | Mouse |
| *Foxa3* | F-CTACATGACCTTGAACCCACTC, R-GGGCTACATACCCGGAAGC | Mouse |
| *Foxa2* | F-CCCTACGCCAACATGAACTCG, R-GTTCTGCCGGTAGAAAGGGA | Mouse |
| *Foxa1* | F-ATGAGAGCAACGACTGGAACA, R-TCATGGAGTTCATAGAGCCCA | Mouse |
| *Foxc1* | F-CGCAGCCCAAGGACATGGTG, R-GATGCCGTTCAGGGTGATCTTC | Mouse |
| *Foxs1* | F-GTTATGGCGGATGCTGTTTTG, R-CTATCCAGAGTTCACCGGGTC | Mouse |
| *Foxb1* | F-TTCCTACAGCGACCAAAAGCC, R-CCGAGGGATCTTGATGAAACAG | Mouse |
| *Foxb2* | F-TTCCTACAGCGACCAAAAGCC, R-CCGAGGGATCTTGATGAAACAG | Mouse |
| *RGMA* | F-CCTCAGGACTTTCACCGACC, R-CGTTCTTAGAGCCATCCACGAA | Human |
| *SLFN5* | F-GAGTGTGTTGTAGATGCAGGAA, R-ACTGCTCGCAGGATGATTTCA | Human |
| *COL15A1* | F-CTGCCCTCGTCCGTATCCT, R-CTGATGGCGAAGTCCCTGA | Human |
| *CHL1* | F-ATGGAGCCGCTTTTACTTGGA, R-GGCAACTTGGACTTTTGACTGT | Human |
| *B ACTIN* | F-AGAGCTACGAGCTGCCTGAC, R-AGCACTGTGTTGGCGTACAG | Human |
| *FOXC1* | F-CGCAGCCCAAGGACATGGTG, R-GATGCCGTTCAGGGTGATCTTC | Human |

Supplementary Table 2. ChIP-qPCR primer sequences

| Name | Sequences | ChIP Ab |
|---|---|---|
| *Grb10* | F- GGGTTCTCGGTTATAGTGTGGGGACTTC<br>R-GGAACTAAGCTGATTCATGTGCCTCCAG | H3K27me$^3$, Foxc1 |
| *Rgma set 1* | F- GATGGTATTTCGGTGCCTCG<br>R- CTGTCCGCGCTTACCTTG | H3K27me$^3$ |
| *Chl1 set 1* | F- GAGGGAGGTGGAAGGAAATC<br>R- AAATCCAGCCTTAGCCACCT | H3K27me$^3$ |
| *Slfn5 set 1* | F- GCAGACCAAAGAAGCAGGTC<br>R- ATTTCCCAGGCACGACAAAC | H3K27me$^3$ |
| *Col15a1 set 1* | F- CACAAGGCAAGCGATGAGAA<br>R- CCCTCACTGTTCACTGGCTA | H3K27me$^3$ |
| *Foxc1* | F- GCCCGAACTCTCTAGCTCTT<br>R- GATGGTATTTCGGTGCCTCG | H3K27me$^3$ |
| *HoxA11 set 1* | F- CCCTTCTCGGCGTTCTTGTC<br>R- CTATAGCACGGTGGGCAGGAAC | H3K27me$^3$ |
| *HoxA11 set 2* | F- CCAGGTTCCAACACAGCCATTC<br>R- CCCATGTGACTCTTTCTAGTCTCCCTTG | H3K27me$^3$ |
| *Rgma set 2* | F- TCCACAGAGAAGCGTTTGTTC<br>R- ACAGACCGGGGATTTAAGCT | Foxc1 |
| *Chl1 set 2* | F- TGCTCTTGCTGGTGGTATTCT<br>R- AGAGAAGATCTTTCCTCTCAGTACA | Foxc1 |
| *Slfn5 set 2* | F- TGTCTGTCGGTGAATGCTCT<br>R- CACCATTGACTGAGGCAGAA | Foxc1 |
| *Col15a1 set 2* | F- GGAGATGGGAGTTGAAGAAGG<br>R- CCCAACGGTACAGGAAACAC | Foxc1 |

Supplementary Table 3. Probes used for Kaplan Meier Survival Curves

| Gene | Probe ID # |
|---|---|
| *FOXC1* | 213260_at |
| *RGMA* | 223468_s_at |
| *DBC1* | 205818_at |
| *SLC9A9* | 227791_at |
| *PDE3B* | 222317_at |
| *CHL1* | 204591_at |
| *DOCK8* | 232843_s_at |
| *SLFN5* | 238430_x_at |
| *COL15A1* | 203477_at |
| *CHN1* | 212624_s_at |
| *RASGRP1* | 205590_at |
| *SLC35F2* | 218826_at |
| *COL4A6* | 213992_at |
| *PCDHB16* | 232099_at |
| *LTBP4* | 204442_x_at |

## Supplementary References

1. Mertins P, Mani DR, Ruggles KV, et al. Proteogenomics connects somatic mutations to signalling in breast cancer. *Nature*. 2016;534(7605):55-62. doi:10.1038/nature18003.
